# Digitalis in Pneumonia, and Salicin in the Hemorrhage of Typhoid Fever

**Published:** 1876-07

**Authors:** 


*Digitalis in Pneumonia, and Salicin in the Hemorrhage of Typhoid Fever.*—Dr. Q. C. Smith (*Pacific Medical and Surgical Journal*) says: When we have a patient suffering from some acute pulmonary affection, attended with high grade of fever, and more especially if the patient is delirious, we often give half to one grain powdered digitalis with three to six grains of lupulin every three to six hours, until the fever abates and the delirium subsides. We direct the nurse to watch the pulse closely, and should it get down to fifty beats per minute, to withhold the medicine until the pulse grows more rapid. This course of treatment will often produce very gratifying results in a few hours. After the more active symptoms are overcome, we frequently add quinine to the prescription.

When we have a typhoid fever patient that is passing blood from his bowels, which symptom is generally accompanied with diarrhœa, we give one to two grains of salicin, with three to six grains of sub. nit. bismuth, every two, four or six hours, until hemorrhage and diarrhœa are checked. We repeat this course as often as the symptoms demand it.

We have found this course to produce better results than the astringents and opiates, as directed by classical authorities, whose teachings we highly respect.

